# Parent–child interaction frequency: associations with age, sibling presence, and child health

**DOI:** 10.1038/s41390-024-03727-x

**Published:** 2024-11-14

**Authors:** Kira Twork, Juliane Ludwig, Wieland Kiess, Tanja Poulain

**Affiliations:** 1 Department of Women and Child Health and Center for Pediatric Research (CPL), Leipzig University Hospital for Children and Adolescents, Leipzig, Germany; 2 LIFE Leipzig Research Center for Civilization Diseases, University of Leipzig, Leipzig, Germany

## Abstract

**Objectives:**

Parent-child interaction plays a crucial role in child development. This study investigated associations between the frequency of parent-child-interactions and sociodemographic characteristics (age, sex, socio-economic status, family structure, number and age of siblings), physical and psychological symptoms in children, and mental health of parents.

**Methods:**

The frequencies of 11 different parent-child interactions (shared reading, singing, moving, painting, building, puzzle, playing ball, role games, language games, number games and talking about problems) were assessed in 739 children aged 2–6.5 years-old using a standardised parental questionnaire, within a population-based cohort study in Leipzig, Germany. Physical and psychological symptoms were investigated using the HBSC Symptom-Checklist and parental depression symptoms using the Patient Health Questionnaire. We applied regression analyses to assess associations between variables.

**Results:**

Shared reading was the parent-child interaction reported most frequently, with an average occurrence of several times a week. Number games were reported least frequently, with an average occurrency of every two weeks. Fewer parent-child interactions were significantly associated with higher child age, higher number of siblings, presence of older siblings and a lower level of physical and psychological symptoms. The other variables (sex, SES, living situation, presence of younger siblings or both (younger and older), depression symptoms of parents) were not significantly associated with the frequency of parent-child interactions.

**Conclusion:**

The findings show that age of children and number as well as age of siblings at home may shape the frequency of parent-child interaction in preschool children. In addition, the findings suggest that parent-child interaction might be related to the health of children rather than the (mental) health of parents.

**Impact:**

- This study assessed the associations between the frequency of different types of parent-child interactions and sociodemographic as well as health-related parameters in a large sample of children.
- There still exist sex-specific differences in the frequency of parent-child interactions related to traditional role models of girls and boys.
- A higher frequency of parent-child interactions is associated with more physical and psychological symptoms in children.
- Parent-child interactions are less frequent in families with more children, especially when older siblings are present.

## Introduction

Interactions between children and their parents shape children’s development and learning, the parent-child relationship, and the child’s personality.^1–3^ Parent-child interactions allow parents to communicate more intensively with their children, which can strengthen their emotional bond.^4–6^

Parent-child interaction is understood as both the quantity/frequency of time spent together^3,7^ as well as the quality of interactions.^1,8,9^ Our primary focus is to investigate the frequency of specific activities parents engage in with their children. Therefore, we define parent-child interaction as the frequency of different activities that primary caregivers share with their child.

Studies show that frequent parent-child interactions such as playing,^3,7^ shared reading,^10,11^ or movement play^12–14^ are associated with better cognitive abilities, language skills, social-emotional abilities, and motor skills of children. In addition, a higher frequency of parent-child interactions is related to fewer behavioural and relationship problems between parents and child.^3,10^ Therefore, it is important to understand what factors may influence the frequency of time parents and children spend together.

Previous studies show that the frequency of parent-child interactions is associated with sociodemographic characteristics. Regarding age, a previous study observed less frequent parent-child interaction in older compared to younger children.^3^

With respect to sex, several studies showed that children prefer to play with toys that are either typical for their sex or sex neutral. These preferences could be influenced by parental role models and mediated through parent-child interaction.^15,16^ Typical play activities for girls include caring for dolls, cooking, painting, and crafting, while playing with cars or weapons and sports activities are more typical for boys.^17^ Furthermore, it was shown that girls share their emotions and talk to their parents about problems more often than boys.^18^

Concerning the family’s social position, a lower socioeconomic status (SES) and a lower education of the parents were shown to be negatively associated with the frequency of parent-child interaction.^1,2,9,19,20^ A study from the US showed that parents with few financial resources have less time for creative shared playtime.^4^

Regarding the children’s living situation, it was found that single fathers engaged more frequently in activities with their children than fathers in two-parent families, while single mothers spent as much time with their children as mothers in two-parent families.^21,22^ In contrast, other studies showed that single parents participate less frequently in family’s leisure activities^23^ and that children are less likely to engage in activities with their mothers if the mother lives without a partner.^9^

The dilution hypothesis by Blake,^24^ which has been confirmed in various other studies,^25–27^ also refers to the living situation of children. It assumes that an increase in the number of siblings leads to a dilution of resources, resulting in diminished educational attainment for the individual child. This dilution includes the time, energy, and attention that parents give to each child^28^ and, therefore, also the frequency of parent-child interaction.

It was also observed that the position within the sibling order may have an impact on the quality of parent-child interactions, with younger siblings receiving less attention than older ones.^29^

Besides sociodemographic characteristics, the health of children and parents was also shown to be associated with the frequency of parent-child interaction. Mental illness or psychological distress in parents are associated with a high degree of perceived parental stress, which might affect the parent-child interaction.^9,19^ Previous studies showed that poorer mental health in parents is associated with poorer parent-child interaction^20^ and that children of affected parents more often show dysfunctional social behaviour.^30^ Regarding the relationship between mental health of children and parent-child interaction, it has been shown that children who experience more neglect from their parents more frequently show psychosomatic symptoms.^31,32^ On the other hand, more frequent communication between parents and their children and a high level of care are associated with a reduced prevalence of mental health problems^33^ and improved coping strategies in children.^18^ In addition, a weak ability to express emotions, which is also learned from interactions with parents,^18^ is recognised as a risk factor for the development of psychosomatic symptoms.^32^ These psychosomatic symptoms often manifest themselves in the form of physical or psychological symptoms such as pain, sleep problems, or concentration difficulties.^31,32^

The present study investigates associations between the frequency of parent-child interactions and different sociodemographic and health-related characteristics of the children and their parents. While previous studies in this field often focused only on a few activities or domains of interactions,^8,11,12,14,18^ we assessed a wide range of potential parent-child interactions including different types of joint play but also time spent on speaking about problems. Based on previous studies, we hypothesised that a lower SES, higher child age, living in family forms other than with two biological parents, higher number of siblings, presence of older siblings, higher number of physical and psychological symptoms of children, and parental depression symptoms are associated with a lower frequency of parent-child interactions.

## Materials and methods

### Subjects

The data analyzed in the present project were collected between 2017 and 2022 within the LIFE Child study. LIFE Child is a population-based cohort study of healthy children (not suffering from any chromosomal or syndrome diseases) based at the Leipzig Research Center for Civilisation Diseases at Leipzig University, Germany. The study investigates the association between health and physiological development of children and internal as well as external influencing factors.^34,35^

Parents gave informed and written consent for their children to take part in the study. The study protocol was approved by the Ethics Committee of Leipzig University (Reg. No. 477/19-ek) and complies with the guidelines of the Declaration of Helsinki.

The following analysis included all healthy children aged between 2 and 6.5 years whose parents had answered the questionnaire about the frequency of parent-child-interaction.

In the case of participation from siblings we only included the youngest child of the family in our analysis. For children who participated more than once in the LIFE Child study, we included only one randomly selected visit. The final sample included 739 children (47.5% female, mean age 3.73 years-old).

SES was recorded using a SES composite score based on information on parental education, parental occupation, and household equivalent income. The score is adapted to a SES score used in another national, population-based study.^36^ The score ranges between 3 and 21, with higher scores indicating higher SES. Based on specific cut-offs, the SES composite score can be used to categorise the SES of a family as high, medium or low. In a representative sample, the distribution should be 20%–60%–20%.^37^ In the present sample the distribution of SES was 3.4%–27.7%–68.9%.

The number of siblings was assessed through a questionnaire and divided into 3 groups. Group one included children with no siblings (*n* = 236 (31.9%)), group two children with one sibling (*n* = 326 (44.1%)) and group three children with two or more siblings (*n* = 177 (24.0%)). With regard to the age of the siblings, we created four groups, namely younger siblings (*n* = 129 (17.8%)), older siblings (*n* = 304 (44.1%)), younger and older siblings (*n* = 57(7.9%)) and no siblings (*n* = 236 (32.5%)). We excluded twin siblings (*n* = 13) in this grouping and the corresponding analyses.

We divided the living situation of the children into two groups. The first group (two biological parents) included all children living with both biological parents at the time of the survey (*n* = 654 (88.5%)). The second group (other family form) included children who lived either with their biological mother/father and a new partner or with single mothers/fathers (*n* = 85 (11.5%)).

### Measurements

#### Frequency of parent-child interactions

The questionnaire about the frequency of parent-child-interactions was answered by the children’s parents. It investigated 11 types of activities between children and the primary caregivers (usually the parents) in their household (shared reading, singing, moving, painting, building, puzzle, playing ball, role games, language games, number games and talking about problems). In the present context, the act of “talking about problems” entails articulating emotions and anxieties, addressing conflicts, and collaboratively seeking resolutions.

For each activity, parents chose between 6 answer categories (“never” = 0, “once/month” = 1, “every 2 weeks” = 2, “once/week” = 3, “more often than once/week” = 4, “daily” = 5). The items were combined to a total interaction-score ranging from 0 to 55 points, which higher scores indicating a higher frequency of overall parent-child-interaction.

The questionnaire is a short form of the German “Questionnaire on Preschool-Aged Childrens’ Activities in the Family” (Roßbach, H. G. & Leal, T. B., Mütterfragebogen zu kindlichen Aktivitäten im Kontext des Familiensettings (AKFRA). Deutsche Fassung des Questionnaire on Preschool-Aged Children’s Activities in the Family, 1993, unpublished manuscript). In the present sample, Cronbach’s alpha was 0.76, which indicates a good internal consistency of the questions.

#### Physical and psychological symptoms of children

Children’s physical and psychological symptoms were assessed using the Health Behaviour in School-aged Children (HBSC) Symptom Checklist^38^ and was completed by the participants’ parents. It records the frequency of 8 symptoms within the last 6 months, namely headache, abdominal pain, back pain, dizziness, problems with falling asleep, depression, irritation, and nervousness. Answers were given on a 5-point scale ranging from 0 to 4 (“never” = 0, “nearly once/month” = 1, “nearly once/week” = 2, “more often than once/week” = 3, “nearly every day” = 4). Here, we assessed how many of the 8 symptoms had occurred within the last 6 months (number of items not answered by “never”) This score, ranging from 0 to 8, was used for further analysis.

### Depressive symptoms of parents

Parents’ depression symptoms were assessed using the depression scale of the Patient Health Questionnaire.^39^ It records the frequency of 9 symptoms within the last 2 weeks (loss of interest, sadness, sleep problems, lack of energy, loss/increase of appetite, reduced self-esteem, difficulties with concentrating, restlessness/slowing down, suicidal thoughts). For each item, parents chose between four response categories (“never” = 0, “on single days” = 1, “on more than half of the days” = 2, “almost every day” = 3). The individual answers were combined to a total score ranging between 0 and 27 points, with higher scores indicating more symptoms of depression. We analysed the data for mothers (*n* = 182) and fathers (*n* = 163) separately.

### Data analysis

The data analysis was performed with “R” version 4.2.1. Data were described as means and standard deviations (for continuous variables) and absolute and relative frequencies (for categorical variables).

Associations between the interaction-score (dependent variable) and characteristics of the child (age, sex, physical and psychological symptoms) as well as the environment (SES, number and age of siblings, living situation, depression symptoms of mothers and fathers) were analyzed using linear regression analyses. Except for age and sex (which were included as covariates in all models), all independent variables were included in separate models.

In a moderator analysis, all models were checked for interactions between the independent variables and age or sex. Only interactions reaching statistical significance (*p* < 0.05) were presented.

Data was collected partly before (*n* = 653 (88.4%)) and partly during the COVID-19-pandemic (*n* = 86 (11.6%)). Therefore, we assessed the association between time point (before or after 03/2020) and total interaction score using linear regression analysis adjusting for child age and sex. The association was not significant (beta = 0.38 (95% CI −1.32 to 2.08), *p* = 0.66). Therefore, time point was not included as covariate in the other models.

Strengths of associations were described using non-standardised (beta) and standardised (ß) regression coefficients. The significance level was set to α = 0.05.

Associations between the independent variables and the frequency of the individual parent-child activities were assessed using ordinal regression analyses. The results of these analyses are presented in Supplement 1.

In our second supplement analysis, we investigated the association between the parent-child interaction score and the individual physical and psychological symptoms (never versus ever). The results of these regression analyses are presented in Supplement 2.

## Results

### Description of the study population

The distribution of sex, SES, siblings and living situation in the sample as well as the mean values (standard deviation) for the parent-child interaction score, the score of physical and psychological symptoms of the child and depression symptoms of mothers and fathers are presented in Table 1.

**Table 1:** Distribution of sex, SES, siblings and living situation and means (standard deviation) of parent-child interaction score, physical and psychological symptoms from children as well as depression symptoms of parents.

	Possible Range	*n* = 739
Sex:		
female		351 (47.5%)
male		388 (52.5%)
SES:		
low		25 (3.4%)
medium		205 (27.7%)
high		509 (68.9%)
Number of siblings:		
no siblings		236 (31.9%)
one sibling		326 (44.1%)
two or more siblings		177 (24.0%)
Age of siblings:		
younger siblings		129 (17.5%)
older siblings		304 (41.1%)
younger and older siblings		57 (7.7%)
twin siblings^a^		13 (1.8%)
no siblings		236 (31.9%)
Living situation of child^b^:		
two biological parents		654 (88.5%)
other family form		85 (11.5%)
Parent–child interaction score^c^	0–55	39.70 (7.64)
Physical and psychological symptoms^d^	0–8	1.94 (1.50)
Depression symptoms mothers	0–27	3.82 (3.28)
Depression symptoms fathers	0–27	2.67 (3.09)

^a^ We excluded twin siblings in the corresponding analyses.

^b^ two biological parents = living with two biological parents, other family form = living with biological mother/father and a new partner or with single mother/father.

^c^ Sum of the individual point values of each of the 11 activities.

^d^ Sum of the number of symptoms with a proficiency not equal to 0.

**Fig. 1:**
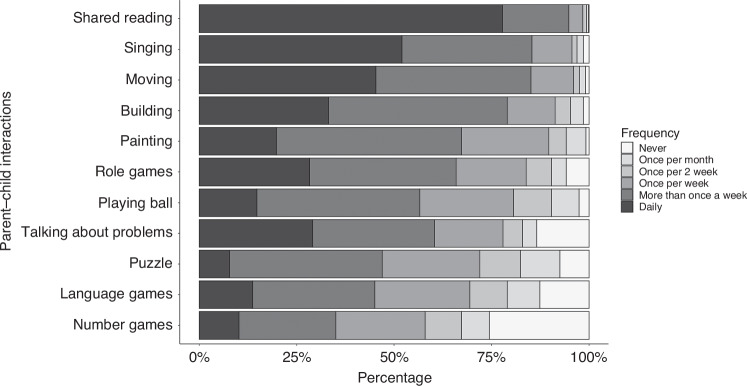
Frequency of the individual parent-child interactions. Bar plot illustrating the distribution of the frequency of the individual activities of the parent-child interaction.

The average parent-child interaction score was 39.70, meaning that, on average, each activity was done once a week. The most frequent activities were shared reading, singing, moving-based play and building, which, on average, took place more often than once a week. Painting, ball games, role games, language games, puzzle and talking about problems were reported on average once a week, and number games every two weeks. The distributions of the individual activities are also shown in Fig. 1.

While male and female sex was equally distributed, we observed an overrepresentation of two-child families (44.1%) compared to families with one (31.9%) or three and more children (24%). Additionally, the group with older siblings (41.9%) was overrepresented compared to the groups of only younger (17.8%) or younger and older siblings (7.9%). Furthermore, the distribution of family structures was also clearly shifted towards families with two biological parents (88.5%) compared to other family forms (11.5%). We also noticed an underrepresentation of families with lower SES (3.4%) and an overrepresentation of families with high SES (68.9%) in our study population.

### Associations between parent-child-interaction score and sociodemographic characteristics, physical and psychological symptoms of the child, and depressive symptoms of parents

The analyses revealed a significant association between higher child age and fewer parent-child interactions (beta = −0.93; *p* < 0.001). In contrast to child age, sex, SES and living situation were not significantly associated with the interaction score (see Table 2). However, some of our additional analyses of the frequency of the individual interactions showed a significant association with sex (see Supplement 1). Painting (OR = 0.42, *p* < 0.001) and singing (OR = 0.74, *p* < 0.05) were more common with girls, whereas building (OR = 2.90, *p* < 0.001) and ball games (OR = 1.93, *p* < 0.001) were more common with boys (see Supplement 1).

**Table 2:** Associations between parent-child interaction score and sociodemographic characteristics, physical and psychological symptoms of the child, and depressive symptoms of parents: non-standardised (beta) and standardised (ß) regression coefficients (+95% confidence interval).

Independent Variables	beta (95% CI)	ß (95% CI)	p
Sex: female^a^	−0.06 (−1.15 to 1.02)	−0.00 (−0.08 to 0.07)	0.908
Age	−0.93 (−1.30 to -0.57)	−0.18 (−0.25 to −0.11)	<0.001***
SES	−0.05 (−0.22 to 0.13)	−0.02 (−0.09 to 0.05)	0.606
Frequency of siblings^b^			
One sibling	−1.84 (−3.13 to −0.55)	−0.12 (−0.20 to −0.04)	0.005**
Two or more siblings	−2.33 (−3.82 to −0.85)	−0.13 (−0.21 to −0.05)	0.002**
Age of siblings (*n* = 726)^b^			
Older siblings	−3.11 (−4.37 to −1.84)	−0.20 (−0.28 to −0.12)	<0.001***
Younger siblings	0.76 (−0.88 to 2.40)	0.04 (-0.04 to 0.12)	0.362
Younger and older siblings	−1.86 (−4.02 to 0.30)	−0.07 (−0.14 to 0.01)	0.091
Living situation: other family forms^c^	0.48 (−1.27 to 2.24)	0.02 (−0.05 to 0.09)	0.589
Physical and psychological symptoms^d^	1.48 (0.41 to 2.56)	0.29 (0.08 to 0.0)	0.007**
Depression symptoms mother (*n* = 182)	−0.16 (−0.54 to 0.21)	−0.06 (−0.20 to 0.08)	0.391
Depression symptoms father (*n* = 163)	−0.19 (−0.57 to 0.18)	−0.08 (−0.23 to 0.07)	0.310

All independent variables were included in separate models, adjusting for child age and sex.

*p < 0.05; **p < 0.01; ***p < 0.001.

^a^ reference = male.

^b^ reference = no siblings.

^c^ reference = two biological parents.

^d^ significant interaction with age, decreases −0.35 per additional year (*p* = 0.006).

Regarding siblings, parent-child interactions were reported significantly less frequently in children with one sibling (beta = −1.84, *p* = 0.005) and two or more siblings (beta = −2.33, *p* = 0.002) than in children without siblings. Furthermore, we observed significantly fewer parent-child interactions when older siblings were present than when no siblings were present (beta = −3.11, *p* = < 0.001). In contrast, the differences between children with no siblings and those with only younger siblings (beta = 0.76, *p* = 0.362) or younger and older siblings (beta = −1.86, *p* = 0.091) were not statistically significant.

Regarding the health of children, a higher frequency of parent-child interactions was significantly associated with more physical and psychological symptoms (ß = 1.48; *p* = 0.007). The moderator analysis showed an interaction with the child’s age. The effect was reduced by −0.35 for each additional year. As revealed by our more detailed analyses (see Supplement 2), a higher frequency of parent-child interactions was significantly associated with some of the individual physical and psychological symptoms in children namely headache (beta = 9.09; *p* < 0.05), abdominal pain (beta = 4.38; *p* < 0.01), depression (beta = 6.06; *p* < 0.01), and nervousness (beta = 7.56; *p* < 0.01) of the child (see Supplement 2).

Regarding depressive symptoms of mothers (beta = −0.16; *p* = 0.391) as well as fathers (beta = −0.19; *p* = 0.310), the analysis revealed no significant association with the frequency of parent-child interactions. The strengths of the associations are also shown in Table 2.

## Discussion

This study investigated the frequency of parent-child interactions in 2 to 6.5 year-old children and associations with sociodemographic and health-related characteristics of children and their parents.

The most frequent parent-child interaction in the present sample was shared reading, which, on average, took place more often than once a week. A possible explanation for this finding is that reading is an evening ritual in many German families that is perceived as something fun and enjoyable, and creates closeness to the child.^11^

Number games took place least frequently, with an average occurrence of every two weeks. This activity requires a lot of patience and concentration and, therefore, might be less popular among children and parents.

### Associations between the frequency of parent-child interactions and sociodemographic characteristics

In accordance with our hypothesis and other study findings, older child age was associated with a significantly lower number of parent-child interactions.^3,40^ One possible explanation for this phenomenon is that older children organise their daily routines more independently from their parents and share more interactions with peers than with parents. The development of their own ideas and goals with increasing age could reinforce this effect of independence.^3,40^

Sex of children showed no significant association with the overall frequency of parent-child interactions. However, our additional analyses revealed significant associations between sex and the frequency of specific activities. Specifically, parents were more likely to paint and sing with their daughters and more likely to build and play ball with their sons. This is also consistent with previous study results and might be explained by traditional role concepts, which are still anchored in our society today.^15–17^

In contrast to other studies,^1,2,9,19,20^ we observed no association between the frequency of parent-child interactions and SES, possibly due to the uneven distribution of SES in our sample. It is also possible that parents of all SES share the same number of activities with their children. Moreover, we only assessed whether parents and children engaged in an activity but not how long. There might have been differences in the duration between families of different SES.

Regarding family patterns, we found no significant association between family form (with two biological parents versus others) and the frequency of parent-child-interactions. Previous studies showed different associations between family constellations and the frequency of parent-child interaction. Some of them are in line with our findings^21,22^ and assume that single parents reduce time for their own needs in favour of time spent with the children.

Regarding the number of siblings present, we observed fewer parent-child interactions in families with two and more as compared to only one child. The results also suggest that it is primarily the presence of older siblings that is associated with lower parent-child interaction. These findings are consistent with the dilution hypothesis and are supported by previous studies.^25–28^ According to the dilution hypothesis, parents divide their time between the children and, thus, have less time for each individual child. This lack of time is probably particularly noticeable for younger children, as they have had to share their parents’ time with other children from the very beginning. Older siblings, on the other hand, may already have established patterns of interaction with their parents that continue even after the arrival of a new sibling.

### Associations between the frequency of parent-child interactions and health of children and parents

In addition to sociodemographic characteristics, we also investigated whether parameters of child physical and psychological health and parental mental health are associated with the frequency of parent-child interaction. In our sample, a higher number of parent-child interactions was significantly related to more physical and psychological symptoms of the child, especially headache, abdominal pain, depression, and nervousness. One explanatory assumption would be that parents who interact more frequently with their children are more likely to notice their physical and psychological symptoms. On the other hand, it is conceivable that children with more physical and psychological symptoms ask more frequently to interact with their parents, e.g., because they are unable to go to kindergarten or to play with peers. Physical and psychological symptoms in children place a high burden on the affected children and their families.^31,32^ Parents may try to alleviate this burden by increasing parent-child interactions.

Another explanatory framework posits, as evidenced by various studies,^32,41–43^ that there exists an association between elevated levels of parental focus on their children’s complaints and the subsequent increase in both physical and psychological symptoms among these children. It appears plausible that parents may inadvertently exacerbate their children’s symptoms through heightened responsiveness to them. Future studies may assess the frequency of parent-child interactions during periods of illness to better understand parental perceptions of children’s physical and psychological symptoms and the possible amplification of these symptoms by parental responses.

Surprisingly, and contrary to previous findings,^9,19,20,30^ no significant association between the frequency of parent-child interaction and the number of parental depression symptoms (for mothers as well as fathers) was found in our analysis. One possible explanation could be the generally low amount of depression symptoms in our sample. This may be due to response biases (social desirability) or the fact that parents with depressive symptoms are less likely to participate in a study.

Furthermore, our study only included healthy children. It is possible that the distribution of physical and psychological symptoms in children from clinical samples would be significantly higher. In this regard, the prevalence of depressive symptoms among parents could also be elevated, as several studies have established a correlation between these two variables.^31,32^ Similarly, within such clinical populations, it is possible that a different association may be observed between the children’s symptoms or the parents’ mental health, and the frequency of parent-child interactions.

### Limitations

All data for our analyses were based on parental perceptions reported in questionnaires and are thus prone to different biases (e.g., social desirability). Furthermore, the questionnaire about the frequency of parent-child interaction did not ask about the duration in time, the quality of the interaction, or the person conducting the interaction (mother, father or, in individual cases, also stepparents or grandparents). Another limitation is the underrepresentation of families with lower SES and the over-representation of families with high SES, which makes our sample not representative for the German population. In addition, we applied a cross-sectional design, making it difficult to draw conclusions on causality.

## Conclusions

The findings suggest that child age and the number and age of children at home are associated with the frequency of parent-child interaction in preschool children, while family socio-economic status and living situation of the child are not. In addition, our results suggest that age and health of the child might show a stronger link to the frequency of parent-child interaction than the (mental) health of parents, at least in families with generally low depression scores.

## Supplementary information

Supplement 1

Supplement 1

## Data Availability

The datasets generated and/or analysed during the current study are not publicly available due to ethical restrictions. The LIFE Child study is a study collecting potentially sensitive information. Publishing datasets is not covered by the informed consent provided by the study participants. Furthermore, the data protection concept of LIFE requests that all (external as well as internal) researchers interested in accessing data sign a project agreement. Researchers who are interested in accessing and analysing data collected in the LIFE Child study may contact the data use and access committee (forschungsdaten@medizin.uni-leipzig.de).
