# Supplementary material for: Parent–child interaction frequency: associations with age, sibling presence, and child health
Source: Pediatr Res. 2024 Nov 14;98(1):114–20. doi: 10.1038/s41390-024-03727-x (PMC12411232; doi:10.1038/s41390-024-03727-x)
Supplement: Supplementary file 2 — Supplement 1 [file 41390_2024_3727_MOESM2_ESM.pdf]

Supplement 2: Associations between the parent-child interaction score and the individual physical and psychological symptoms of the child: non-standardized (beta) and standardized (ß) regression coefficients (+ 95% confidence interval).

Physical and psychological symptoms[a]	Frequency occurrence never (%)	Parent-child interaction score		
		beta (95% CI)	ß (95% CI)	p
headache	92.5	9.09 (1.47 to 16.72)	0.31 (0.05 to 0.57)	0.020*
abdominal pain	63.1	4.38 (-1.08 to 7.68)	0.28 (0.07 to 0.48)	0.009**
back pain	97.3	1.71 (-1.66 to 5.08)	0.04 (-0.04 to 0.11)	0.319
dizziness	98.6	-3.10 (-7.83 to 1.63)	-0.05 (-0.12 to 0.02)	0.199
problems falling asleep	43.4	-0.05 (-1.06 to 1.17)	0.00 (-0.07 to 0.08)	0.925
depression	85.0	6.06 (1.54 to 10.58)	0.28 (0.07 to 0.49)	0.009*
irritation	41.1	-0.33 (-1.44 to 0.77)	-0.02 (-0.09 to 0.05)	0.554
nervousness	85.1%	7.56 (3.06 to 12.06)	0.35 (0.14 to 0.56)	0.010**

[a] reference = never occurring

all independent variables were included in separate models, adjusting for child age and sex

level of significance: *p < 0.05; **p < 0.01; ***p < 0.001
